# Cohort and Case–Control Studies: Strengths, Limitations and Methodological Considerations

**DOI:** 10.1111/jre.70145

**Published:** 2026-07-09

**Authors:** Nasir Z. Bashir, João Botelho, John Rong Hao Tay, Dian Yi Chow, Gustavo G. Nascimento

**Affiliations:** ^1^ MRC Biostatistics Unit University of Cambridge Cambridge UK; ^2^ Egas Moniz Centre for Interdisciplinary Research Egas Moniz School of Health and Science Almada Portugal; ^3^ National Dental Research Institute Singapore National Dental Centre Singapore Singapore Singapore; ^4^ Health Services Research and Population Health Programme Duke‐NUS Medical School Singapore Singapore; ^5^ Department of Restorative Dentistry National Dental Centre Singapore Singapore Singapore; ^6^ School of Dentistry University of Utah Salt Lake City Utah USA

**Keywords:** birth cohort, causal inference, cohort studies, epidemiology, longitudinal studies, periodontitis

## Abstract

Cohort studies are central to modern health research, enabling the investigation of disease incidence, progression and long‐term consequences of exposures. However, cohort designs vary substantially in structure and analytic implementation, and these differences have important implications for their validity and interpretation. This article provides a methodological overview of cohort study design and analysis in periodontology, with emphasis on how design choices and analytical strategies influence the potential sources of bias. We describe the major cohort variants, including prospective, birth, retrospective and nested case–control designs and examine how each shapes exposure measurement, follow‐up and outcome ascertainment. Key design considerations include definition of the source population, specification of time zero, handling of dynamic populations and risk sets and the distinction between fixed and time‐varying exposures. We then discuss major sources of bias relevant to cohort studies, including confounding, selection bias (index event bias and prevalent user bias included), immortal time bias, censoring and competing risks. Design‐based strategies, notably the new‐user design and target trial emulation, are presented. We further clarify the basis of a recurring study type in the periodontal literature—single‐arm treatment series framed as cohort studies—and discuss how these relate to the cohort design and the inferences they support. Finally, we compare cohort and case–control designs, highlighting both their shared methodological foundations and their important differences in sampling, efficiency, interpretation and application. The credibility of a cohort study depends less on the application of any single statistical method and more on the coherence between the research question, the structure of the data and the assumptions required for valid interpretation. Careful design, transparent reporting and explicit acknowledgment of limitations are essential if cohort studies are to support reliable inference in periodontal research and beyond.

## Graphical abstract

Population‐based epidemiology and clinical research are complementary by design—and nowhere is that more evident than in cohort studies. By following individuals over time, cohorts quantify disease incidence, elucidate environmental and social determinants and evaluate biomarkers and prognostic outcomes within a single longitudinal framework. Their architecture underpins translational science, comparative effectiveness, real‐world evidence and precision dentistry.

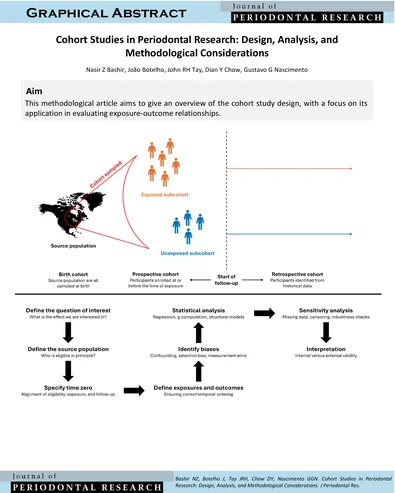

## 1 Introduction

This article provides a dedicated overview of cohort study design and methodology. The article is supplemented with examples and discussion pertaining to periodontology, though the principles we outline apply broadly to clinical research as a whole. Building upon the conceptual framework established in the introductory article of this series, we discuss the defining features of cohort designs, their variants, and the major sources of bias that threaten their validity [1]. Cohort studies in periodontology serve at least three distinct purposes: aetiologic inference, prognosis and prediction modelling. These aims should not be conflated. A design optimised for aetiologic inference may not be optimal for prediction and vice versa. More generally, different study designs and analytical choices correspond to different research questions and target quantities of interest, often termed *estimands* in the causal inference literature. For example, a study may aim to estimate the effect of sustained periodontal exposure over time, the effect of baseline exposure alone, or the effect of initiating a particular treatment strategy, each of which represents a distinct estimand and requires different methodological approaches. Further discussion is available in the causal inference primer within the Methodological Series of this journal [2].

Our focus in this paper is primarily on the use of cohort studies to evaluate exposure–outcome relationships, with a view to elucidating causal effects. Cohort designs also play an important role in evaluating treatment effectiveness and the persistence of treatment effects over time, particularly in periodontology, where allocation to non‐treatment groups may be ethically unacceptable. Other applications of cohort studies, such as their use in predictive modelling and disease surveillance, are important but largely outside of the scope of this discussion but can be found in other epidemiological literature [3]. A comparison of considerations for predictive and inferential modelling is presented in Box 1.

BOX 1 **Comparison between predictive modelling and inferential modelling.**

**Table:** 

**Prediction** Aims to estimate what is likely to happen. Focus is on forecasting outcomes in new individuals or populations. Associations may be very informative for prediction even if they are non‐causal. Model performance is typically evaluated using metrics such as discrimination or calibration. **Example:** Having yellow fingertips (‘smoker's fingers’) may be predictive of periodontitis.
**Causation** Aims to estimate what would happen under a change or intervention. Focus is on understanding whether changing an exposure would change an outcome. Exposures can be strongly associated with the outcome without being causal. Requires assumptions that allow for causal interpretation, particularly like‐for‐like comparisons. **Example:** Smoking cessation may reduce the risk of developing periodontitis in the future.

We also review methodological developments that have improved the credibility of observational cohort evidence. Throughout, published cohort studies in periodontology are discussed to illustrate design principles in real terms. These examples are used not because they are exhaustive, but because they span major design variants, analytical challenges and research questions characteristic of periodontal cohort research. Key terms used throughout this article are provided in Table 1 for reference.

**TABLE 1:** Glossary of key terms.

Term	Definition
Competing risk	An event that either prevents the outcome of interest from subsequently occurring or fundamentally alters the probability that it can be observed. In periodontal cohorts, tooth loss is often a competing risk for measures of periodontal destruction and is rarely a neutral censoring event.
Confounding	A central threat to causal inference in cohort studies. It arises because exposure is not randomly assigned but reflects the complex interplay of biological, behavioural and social factors that may also determine the outcome. This means any observed association is a mixture of the true effect and the contribution of shared determinants.
Directed acyclic graph	A visual framework for encoding causal assumptions, and used to identify the minimal set of variables needed to block confounding paths and to distinguish confounders from mediators and colliders. A directed acyclic graph is only as credible as the causal hypotheses it encodes.
Dynamic (open) versus fixed (closed) population	In a dynamic population, person‐time accrues from a constantly evolving group of individuals who enter and exit over calendar time, as in an electronic health record database. In a fixed population, exposure groups are defined at the start of follow‐up and membership does not subsequently change, as in the arms of most randomised trials.
Immortal time bias	Bias arising when a period during which the outcome cannot occur by design is erroneously included within the follow‐up or exposure window, thereby artificially making the exposure appear protective.
Index event bias	A form of selection bias that can arise when an analysis is restricted to people who already have a particular disease or have experienced a particular event. Restricting the analysis in this way can create apparent associations between the causes of that disease or event, which distorts estimates of what influences its subsequent course.
Person‐time (time at risk)	The period during which an individual is under observation and genuinely susceptible to experiencing the outcome of interest. Only person‐time accrued while participants are both observable and eligible to develop the outcome should contribute to the denominator when estimating disease rates.
Prevalent user bias	Bias arising when the analytical sample includes individuals already using a treatment of interest at the time of study entry. Prevalent users represent a biologically selected group of long‐term survivors who differ systematically from treatment‐naive individuals, distorting comparative estimates.
Risk set	The set of cohort members who, at a given point in follow‐up, remain free of the outcome and eligible to develop it. Defining the risk set at baseline ensures that subsequent outcome events represent true incident cases rather than prevalent disease carried over from before the observation period.
Time‐fixed versus time‐varying	A time‐fixed variable is assumed to remain constant throughout follow‐up, and often measured at baseline, such as place of birth. In contrast, a time‐varying variable can change over the course of follow‐up, such as income or medication use. Properly accounting for time‐varying exposures and covariates is important to avoid bias.
Time zero	The synchronised time point in a study when eligibility criteria are met, treatment strategy is assigned, and follow‐up for the outcome begins. Misspecification of time zero is a common source of immortal time bias.

### 1.1 Conceptual Basis of Cohort Studies

This study design begins by identifying a group of individuals, a *cohort*, who share one or more defining characteristics, and then follows them over time (longitudinally) [3]. Often, this shared characteristic is an exposure of interest. However, the defining characteristic need not be an exposure, and may instead be any feature of the sampled individuals, including geographical location, birth year, occupation or demographic attribute [3]. For example, the 1982 Pelotas Birth Cohort is comprised of individuals born in the Brazilian city of Pelotas with a common birth year of 1982.

Regardless of the shared characteristic(s), cohort studies are very often used to infer the effect of an exposure on a given disease outcome. The benefit of the cohort design is that it establishes temporal ordering; by measuring the exposure before the outcome occurs, the investigator can establish that the putative cause precedes its presumed effect, a necessary (though not sufficient) condition for causal inference [3]. When a cohort study is used to elucidate the effect of an exposure, its design is structurally analogous to the randomised controlled trial (RCT), from which it differs primarily by the absence of random exposure assignment, thereby introducing potential confounding to the exposure–outcome relationship (confounding is discussed in more detail later on).

A potential source of confusion is that the term cohort may be used in two related but distinct ways: (i) to refer to the entire group of individuals under study, or (ii) to refer to a subgroup defined by exposure status. In studies comparing exposed and unexposed participants, one subgroup is often described as the *exposed* cohort, while the other serves as the *unexposed* or *reference* cohort [3]. The reference cohort provides the comparator against which the risk observed in the exposed group is evaluated. Here, we use the term cohort to refer to the study population as a whole, reserving the term *subcohort* for exposure‐defined groups within that population.

## 2 Types of Cohort Studies

### 2.1 Prospective Cohort Studies

In a prospective cohort study, the investigator identifies the cohort at or before baseline, actively measures exposures and covariates at predefined time points, and then follows participants forward in time to ascertain outcomes. Prospective designs offer the greatest control over measurement quality. This is because exposures and covariates are collected using standardised protocols designed specifically for the research question rather than administrative convenience, biospecimens can be banked for future hypothesis‐driven assays, and outcomes can be systematically adjudicated against pre‐specified criteria. Importantly, prospective data collection is less prone to *recall bias*, whereby participants misreport past exposures due to imperfect memory or differential recall related to disease status; this represents a distinct advantage of prospective cohort studies compared to retrospective studies.

A useful distinction in cohort studies is between *study time* and *epidemiologic time*. Study time refers to when the investigator assembles the cohort and collects data, whereas epidemiologic time refers to the temporal ordering of exposure and outcome. In epidemiologic time, exposure classification always precedes outcome ascertainment in properly defined cohort studies, regardless of whether the study is conducted prospectively or retrospectively. The distinction between prospective and retrospective designs, therefore, relates to study time, not to the direction of time in the underlying data‐generating process.

The SHIP cohort exemplifies the approach of prospective data collection, with standardised full‐mouth periodontal assessments and biological sample banking at each examination wave [4]. Using this cohort, Demmer and colleagues demonstrated that greater periodontal disease severity at baseline predicted less favourable glycaemic trajectories at 5‐year follow‐up. This was observed among both normoglycemic participants and those with pre‐existing impaired fasting glucose, suggesting that periodontitis accelerates glycaemic deterioration across the metabolic risk spectrum [5]. Subsequent analyses of SHIP have extended these findings to examine the reverse association and to evaluate specific inflammatory mediators as potential pathway variables [6]. In addition, the parallel SHIP‐TREND cohort has enabled internal replication of key associations in an independently recruited sample, a form of consistency check not possible in single‐cohort designs.

Beyond aetiological research, prospective cohort designs are also the primary vehicle for characterising the natural history of disease, including incidence, progression and distribution of trajectories within and across populations. The longitudinal studies of periodontal disease in Norwegian students and Sri Lankan workers by Löe and colleagues exemplify this application [7]. By following populations with markedly different levels of professional dental care over decades and documenting clinical attachment loss at regular intervals, these studies established that periodontal destruction, in the absence of treatment, is near‐universal yet heterogeneous. That is, a minority of individuals experience rapid attachment loss, the majority progress at a moderate rate, and a small proportion show little or no progression despite comparable plaque exposure. This paradigm of differential susceptibility, derived from prospective observation without intervention, shaped subsequent aetiological hypotheses concerning host response and risk modification that continue to inform periodontal research today.

### 2.2 Retrospective Cohort Studies

In a retrospective cohort study, both exposure and outcome information are ascertained from records compiled prior to the formulation of the research question. The term *retrospective* only refers to study time, that is, the timing of data collection and cohort assembly. In epidemiologic time, the temporal ordering still proceeds forward from exposure to subsequent outcome. Investigators define a historical baseline, reconstruct exposure from administrative, clinical or biological records, and ascertain outcomes from subsequent records. Because the data already exist, retrospective cohorts can often be assembled more rapidly and at lower cost than prospective designs, and access to large administrative databases enables sample sizes that would be infeasible in prospective studies.

Kim and colleagues illustrated this approach using the National Health Insurance Service–Health Examinee Cohort, exploring the association between health status and tooth loss in Korean adults [8]. Larger administrative databases (e.g., electronic health records, national health insurance claims registries and primary care research networks) have extended this approach to samples of hundreds of thousands of participants, enabling investigation of associations between periodontal conditions and rare systemic outcomes with sufficient statistical power, as well as subgroup and interaction analyses that are typically infeasible in smaller cohorts [9].

The principal limitation of retrospective cohorts is that the investigator has no control over how the original data were collected. Many increasingly popular data sources, including electronic health records, disease registries and insurance claims databases, were generated primarily for clinical care, administrative documentation or billing purposes rather than for research. Consequently, periodontal diagnoses may be inconsistently recorded across providers, clinical attachment level measurements may be absent or non‐standardised, and important confounders such as dietary habits, oral hygiene behaviours or socioeconomic position may not have been systematically documented. In addition, recall bias may arise when exposure information is reconstructed from patient‐reported histories recorded after the fact. Incomplete clinical documentation can also lead to potential misclassification that is difficult to quantify or correct. These cohorts should therefore include rigorous sensitivity analyses to assess the robustness of findings to violations of key analytic assumptions and deviations from ideal data conditions.

These limitations should not be interpreted as implying that retrospective cohort studies are inherently weaker than prospective cohort studies in all respects. In certain settings—particularly where large‐scale routinely collected data allow near‐complete ascertainment of exposure, follow‐up and rare outcomes—retrospective cohort designs may offer advantages in terms of representativeness, efficiency and statistical precision that are often not feasible in smaller prospective studies.

### 2.3 Birth Cohort Studies

Birth cohort studies represent a particularly important variant of cohort designs. They are defined by sampling at birth in epidemiologic time, but, with respect to study time, they may be conducted prospectively or retrospectively depending on whether follow‐up is ongoing or reconstructed from historical records.

By enrolling participants at birth, they capture life course data that is less vulnerable to recall error and ambiguity of temporal ordering than if the same data were collected retrospectively in adult cohorts. Some exposures of aetiological interest in periodontology benefit particularly from measurement at cohort inception rather than merely preceding the outcome. Socioeconomic position at birth, for instance, is a characteristic of the family environment into which an individual is born, and although it can be asked about in later life, adult recall is shaped by subsequent experience and subject to differential error related to current health status. A report from the 1982 Pelotas Birth Cohort used this feature to estimate the controlled direct effect of early‐life family income on periodontitis in adulthood, that is, the effect of childhood socioeconomic position that operates independently of adult behavioural and socioeconomic pathways [10]. This analysis illustrates the unique methodological advantage of birth cohorts: prospective measurement of early‐life exposures combined with decades of prospectively collected mediator and covariate data allows temporal ordering between exposure, mediators and outcome to be established by design. Such ordering is difficult to reconstruct reliably in adult‐enrolment cohorts, where childhood exposures, adult pathways and current outcomes are often measured concurrently.

The Dunedin Multidisciplinary Health and Development Study extends this design further by combining the structural advantages of the birth cohort with repeated periodontal assessments across multiple adult waves [11, 12, 13]. This enables investigation not only of risk factors for incident disease, where exposures can be characterised prospectively from childhood and adolescence, but also of periodontitis progression within the same individuals over time. Reanalysis of the Dunedin periodontal data has shown that this study design allows key hypotheses regarding exposure–outcome relationships to be revisited, in some cases leading to different conclusions [14].

This capacity to distinguish incidence from progression represents a distinctive strength of birth cohorts with repeated clinical assessments.

### 2.4 Nested Case–Control Studies

A nested case–control study is an efficient sampling strategy that follows a hybrid design, applied within a broader established cohort. Among cohort members who develop the outcome (cases), a sample of members who had not yet developed the outcome at the time of each case's event is selected as controls, matched on time at risk (known as *risk set sampling*). Exposure and covariate information are then assessed for both groups.

Because biological specimens or detailed measurements need only be analysed for a subset of cohort members, this approach substantially reduces cost without sacrificing the temporal ordering and other advantages inherent in the parent cohort. Schulz and colleagues illustrated this design by sampling individuals from within the Kiel Food Chain Plus cohort by analysing stored serum samples for secreted frizzled‐related protein 5 (sFRP5) and Wnt family member 5A (Wnt5a) in a case group of participants with periodontitis and tooth loss and matched controls without tooth loss [15]. Cases had lower circulating sFRP5 concentrations, suggesting that this protein may be a biomarker of periodontitis severity, although this evidence is based on cross‐sectional comparisons rather than disease progression. Investigators may consider designs of this kind as they can generate biologically informative findings while avoiding the expense of biomarker assessment across the full cohort. Its value extends beyond biomarker research; nested case–control studies are useful whenever data are relatively easy to collect or store, but expensive or labour‐intensive to analyse. This includes stored biological specimens requiring complex laboratory assays, but also visually encoded data such as baseline radiographs or cone‐beam computed tomography scans that require manual interpretation, handwritten clinical notes requiring chart review, or electronic records lacking accessible backend extraction tools where variables must be coded manually.

Briefly, the concept of *time at risk*, also known as *person‐time*, refers to the period during which an individual is under observation and genuinely susceptible to experiencing the outcome of interest. Typically, only person‐time accrued while participants are both observable and eligible to develop the outcome should contribute to the denominator when estimating measures such as disease rates or hazards. Correct specification of time at risk is therefore fundamental to valid inference in longitudinal research.

### 2.5 Case‐Cohort Studies

Case‐cohort studies are technically a form of case–control study in which the source population is a well‐defined cohort [3]. In the classical design, a random subcohort is sampled from the full cohort at baseline, such that every eligible cohort member has a known, equal probability of selection, irrespective of future case status. This sample then serves as the comparison group for one or more outcome analyses, where all incident cases arising in the cohort may be included. Because the same subcohort can be reused across multiple outcomes, the design offers substantial efficiency gains when exposures are expensive to measure or when several disease endpoints are of interest. In effect, case‐cohort approaches can be viewed as efficient means of conducting multiple case–control analyses within a single underlying cohort while aiming to preserve a clearly defined source population. We do not consider the case‐cohort design further in this review. It is noted here primarily because its name may suggest that it is itself a full cohort design, whereas in methodological terms it is better understood as a case–control sampling strategy nested within a cohort framework.

The different cohort study designs are illustrated in Figure 1 and Table 2 summarises the key features and trade‐offs of cohort variants.

**FIGURE 1:**
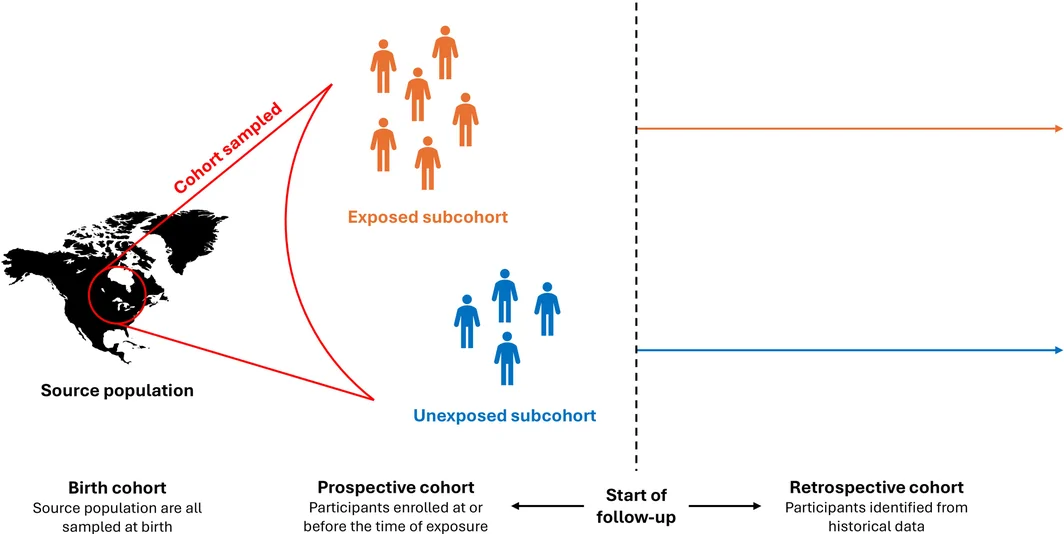
The distinction between prospective and retrospective cohorts lies in study conduct time. Prospective cohorts enrol participants at or before the time of exposure and follow them forward in time. Retrospective cohorts reconstruct exposure and follow‐up using historical records after the relevant events have already occurred at the time of study conduct; however, in epidemiologic time, exposure classification still precedes outcome ascertainment. Birth cohorts involve sampling a cohort consisting of newborns and following participants across the life course. Note that a birth cohort may be followed prospectively or reconstructed retrospectively.

**TABLE 2:** Comparison of study design variants and their key features, with periodontal research examples.

Feature	Prospective cohort	Birth cohort	Retrospective cohort	Nested case–control
Baseline disease‐free requirement	Enforced through eligibility screening at recruitment.	Generally satisfied, as disease does not occur before birth.^a^	Ascertained from existing records, may be incomplete.	Inherited from the parent cohort.
Exposure measurement	Ascertained at or before follow‐up time begins, often with a standardised protocol.	Life course; may be repeated at multiple developmental stages.	Reconstructed from administrative or clinical records.	Often retrospective within parent cohort for example, stored specimens or radiographic measures, but can be collected at a later date.
Time‐varying confounding	Depends on how many measurements were taken. Cohort studies are particularly rich in that they enable g‐computation and structural modelling approaches. Note that retrospective cohorts are usually limited by what was recorded historically.			Inherited from parent cohort design.
Cost and time	High.	Very high due to decades of follow‐up.	Low‐to‐ moderate.	Lower than full cohort analysis.
Periodontal example	SHIP: SHIP‐0 full‐mouth exam, HbA1c trajectory at SHIP‐1/SHIP‐2 [4].	1982 Pelotas Birth Cohort: life course obesity, periodontitis at age 31; Dunedin DMHDS: repeated periodontal exams throughout adulthood, disease progression analysis [16].	Kim et al.: National Health Insurance Service–Health Examination cohort, tooth loss and health status [8].	Schulz et al.: Kiel Food Chain Plus, sFRP5 and tooth loss in patients with periodontitis [15].

Abbreviations: HbA1c, glycated haemoglobin; sFRP5, secreted frizzled‐related protein 5.

^a^ Though generally satisfied, in utero or congenital processes may predate birth, and some cohorts enrol pregnant women to allow for evaluation of such effects.

## 3 Key Design Elements

### 3.1 Source Population and Eligibility Criteria

The source population defines the group from which cohort members are drawn and determines the population to which findings can, in principle, be generalised if the analytic sample is representative. It should be explicitly described in terms of its geographic, demographic and clinical boundaries, but also in terms of the mechanism by which individuals enter that population. In many clinical cohort studies, participants are recruited simply as consecutive patients attending a clinic over a specified period (i.e., a convenience sample). While practical, this often leaves ambiguous what the underlying source population is intended to be. For example, such a sample is very unlikely to represent all individuals with periodontitis in the wider community, but rather the subset who seek care, have access to services, are referred, or present with disease severe enough to attend specialist clinics. Consequently, the sampling process itself may shape the observed distribution of exposures, outcomes and covariates. Assertions of generalisability, therefore, require more than describing the clinic setting—they require a clear statement of the target population. Eligibility criteria then further define the analytic sample within that source population. Restrictive criteria may improve internal validity and reduce loss to follow‐up but limit external validity, whereas broader criteria may improve generalisability at the cost of increased heterogeneity and potential complications in the control of confounding and other sources of bias.

A source population with a high prevalence of key confounders or effect modifiers, restricted access to preventive care, or unusual socioeconomic patterns may yield effect estimates that differ from those in other settings, not because the underlying biology differs, but because shifts in the distribution of these variables alter the estimand. The SHIP and the Pelotas cohorts, drawn from very different populations in terms of socioeconomic context, dietary patterns and oral health infrastructure [4, 16], reflect distinct confounder profiles. Comparisons across such cohorts are, therefore, informative precisely because they test the consistency of associations across varying population conditions.

The presence of edentulism deserves particular attention in periodontal cohorts because its implications depend on the role of periodontitis within the analysis. When periodontitis is the outcome, completely edentulous individuals are no longer at risk of developing incident periodontal disease around natural teeth, and their exclusion is therefore appropriate. However, when periodontitis is treated as the exposure, complete edentulism may create exposure misclassification because the current periodontal status cannot be directly assessed, despite edentulism potentially reflecting a history of severe periodontal destruction. Partial tooth loss presents additional challenges: restricting analyses to individuals with complete dentition or excluding participants with missing teeth may produce a selected sample that does not reflect the full distribution of periodontal disease burden in the source population. Because tooth loss may itself be influenced by periodontitis, caries, smoking, socioeconomic conditions and other shared determinants, such restrictions may also introduce undesirable types of selection bias (i.e., collider bias) [17]. Hence, cases of complete edentulism should be carefully evaluated, and if possible, sensitivity analyses accounting for these individuals should be performed.

Finally, the characteristics of the individuals under study also require consideration. As aforementioned, when calculating rates (e.g., of disease incidence or hazards), the concept of person‐time is central. In many cohort analyses, the amount of follow‐up accrued across participants is therefore more informative than the simple number of individuals enrolled. What matters is not only how many people enter the study, but how long they remain under observation and at risk of experiencing the outcome. This is illustrated in Figure 2 using a hypothetical cohort comparing tooth loss in smokers versus non‐smokers.

**FIGURE 2:**
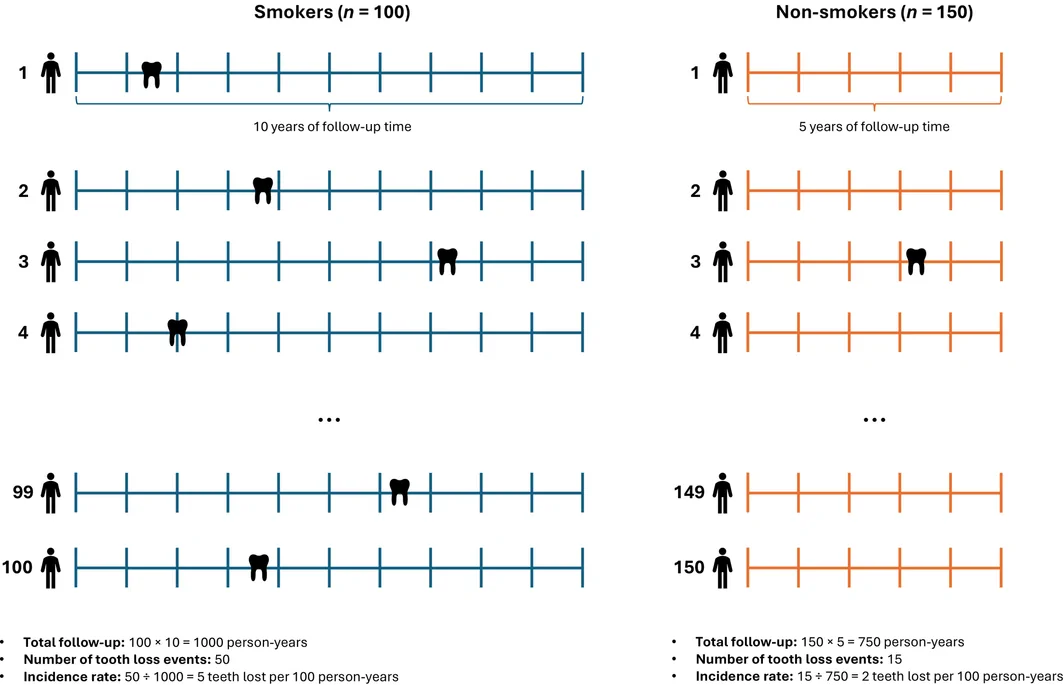
Hypothetical cohort study comparing incidence of tooth loss in smokers versus non‐smokers, where smokers are followed for 10 years each and non‐smokers for 5 years each. Even though more non‐smokers are enrolled, the total amount of follow‐up time contributed by smokers is greater. This is important because it is the follow‐up time that is used to calculate incidence rates. Assuming that 50 teeth are lost among the smokers and 15 teeth among the non‐smokers, this yields a higher rate of tooth loss (per person‐year) among the smokers. The tooth icon denotes a tooth loss event experienced by an individual. For clarity, not all events are shown in the diagram (i.e., 50 events in smokers and 15 in non‐smokers).

This emphasis on person‐time also means that the membership of an exposure group need not consist of the same individuals at every point in time. In a *dynamic*, or *open*, population, person‐time accrues from a constantly evolving group of individuals who enter and exit the population over calendar time. Examples include patients entering and leaving an electronic health record database. The incidence rates derived from such populations reflect the aggregate experience of many individuals, not a single fixed set followed continuously from baseline. By contrast, when exposure groups are defined at the start of follow‐up and membership does not subsequently change, the design involves *fixed*, or *closed*, cohorts. The treatment arms of most randomised trials provide the clearest example. Distinguishing between the type of population under study is important because each has different implications for estimation, inference, interpretation and generalisability of results.

### 3.2 The Risk Set

A key requirement at study entry is that all participants must be free of the outcome of interest, and also at risk of developing it. That is, individuals who cannot develop the outcome of interest are typically not included within the cohort or, at least, not included within the analytic sample. This criterion defines the study's *risk set* at baseline and ensures that subsequent outcome events represent true incident (i.e., new) cases rather than prevalent disease carried over from before the observation period (see discussion of prevalent user bias below). In most adult population‐based cohorts, this condition must be explicitly enforced. The SHIP cohort, for example, excluded participants with a pre‐existing diabetes diagnosis and those with glycaemic values already in the diabetic range at SHIP‐0, thereby restricting the analytic sample to individuals with a non‐zero risk at cohort entry. Failure to enforce this criterion is among the most common errors in establishing a true cohort study and can introduce substantial bias into downstream analyses. This is particularly problematic in electronic health record‐based cohort studies, where prevalent and incident cases may not be clearly distinguished in routinely coded data.

Birth cohorts satisfy this requirement in a structurally distinct and methodologically elegant manner. Because participants are enrolled at birth—or, for outcomes measurable only later in life, at a stage preceding any possibility of disease onset—baseline disease‐free status is guaranteed without the need for explicit eligibility screening. In the 1982 Pelotas Birth Cohort, for instance, all participants were enrolled at birth, and periodontitis, a condition requiring time‐dependent and host‐microbiological interactions to develop, could not have been present at cohort inception (individuals were without dentition upon enrollment into the cohort). This design feature eliminates the risk of prevalent case contamination and removes a source of uncertainty that affects many adult‐enrollment cohorts. Subsequent follow‐up waves provided the first opportunities to observe periodontal outcomes, with the preceding waves providing rich longitudinal data on the life course exposures of interest [16].

### 3.3 Exposure and Outcome Measurement

A defining feature of prospective cohort studies is that exposure is measured before the outcome occurs, and this temporal structure creates methodological challenges that are specific to the longitudinal design. First, exposures may be fixed at baseline or time‐varying, and this distinction has important analytical consequences. The notion of a fixed versus time‐varying variable refers to the fact that some variables are static over time, whereas others change with time. This difference is illustrated in Figure 3. A fixed baseline exposure, such as place of birth, requires a single measurement. A time‐varying exposure, such as periodontal disease status or income, requires repeated assessments and researchers must decide whether to model the exposure at a single time point, average it across follow‐up, or represent it as a trajectory or cumulative measure. Furthermore, exposure may change in a dynamic manner with time; for example, someone may smoke, quit smoking and restart, and there are numerous ways in which this can be handled in downstream analyses. Each specification encodes a different causal question and corresponds to a different estimand that is, the precise effect being estimated, and therefore the question being answered, depends on how exposure is defined over time and how the analysis conceptualises follow‐up. Therefore, the chosen operationalisation should be explicitly justified in terms of the biological mechanism.

**FIGURE 3:**
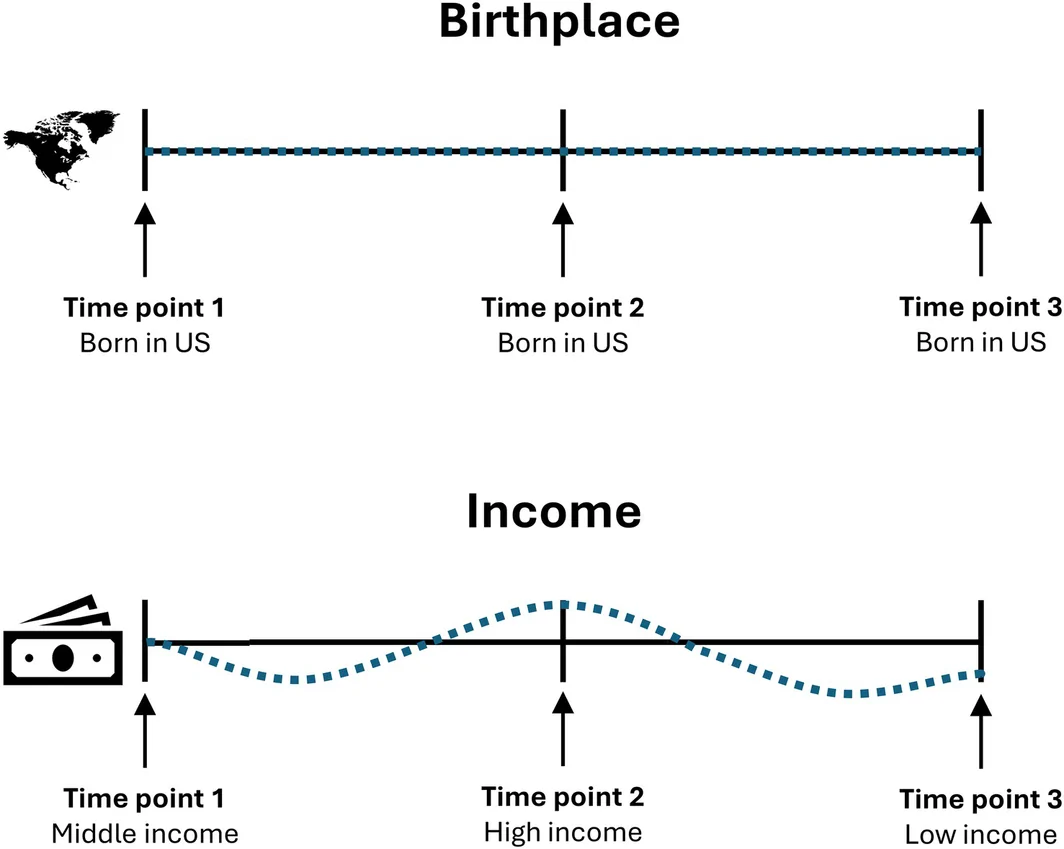
Comparing the variables place of birth and income. Place of birth is determined at birth and cannot vary with time. No matter when it is measured over time, it does not change. Income is not determined at birth and can vary with time. Therefore, the value it takes depends on when it was measured. Consideration of whether a variable is fixed or time‐varying may have implications for downstream analyses.

Second, cohort studies face the problem of exposure misclassification at baseline, and this error may propagate forward in time. Non‐differential misclassification of a binary exposure typically attenuates effect estimates toward the null, but differential misclassification, where measurement error differs by outcome risk, can bias estimates in either direction [3, 18, 19, 20]. Where full‐mouth periodontal examination is not feasible, partial‐recording protocols systematically underestimate disease extent, and the expected degree of attenuation should be estimated and reported alongside primary findings. This can also be investigated systematically using *quantitative bias analysis*, which allows investigators to assess the extent to which their findings are vulnerable to such sources of error [21, 22]. In addition, sensitivity analyses using alternative exposure definitions are particularly informative in cohort studies because the exposure definition also determines which participants are classified as exposed versus unexposed, and therefore which causal contrast is being estimated [10, 23].

Third, for cohort studies examining the effect of treatment, rather than a naturally occurring exposure, the distinction between incident and prevalent treatment is critical. Prevalent users of a treatment at baseline have already survived an initial exposure period, which selects against those who experienced early adverse effects or discontinued treatment. As discussed in the section on the new‐user design below, restricting analyses to individuals initiating treatment for the first time at a well‐defined time zero is essential to avoid this bias, assuming one wishes to emulate the structure of an RCT (please refer to the target trial emulation article in this series [24]).

Outcome definition can face many of the same challenges as discussed above for exposure measurement. Therefore, outcome ascertainment must be pre‐specified, systematic, and applied consistently across exposure groups to minimise differential misclassification. For systemic outcomes, such as incident diabetes, multiple complementary sources are typically combined: laboratory values, medication dispensing records, self‐reported diagnoses and linkage to administrative data, each with different sensitivity and specificity profiles.

For periodontal outcomes, standardised clinical examination protocols with documented examiner training, calibration exercises and reported reliability coefficients represent the reference standard. However, in practice, periodontal outcomes are also frequently ascertained using routinely collected data sources, including dental treatment records, procedural and diagnostic codes in administrative databases, and, in some settings, self‐reported measures of periodontal disease or tooth loss. While these approaches enable large‐scale and resource‐efficient studies, they typically provide lower resolution and greater susceptibility to misclassification compared with full‐mouth clinical examinations. The validity of these approaches depends on the structure of the underlying database, the clinical coding practices in the healthcare system, and the extent to which recorded diagnoses reflect true underlying disease rather than service utilisation or reimbursement processes. Investigators using such outcome measures should therefore consider the extent to which their data source is likely to be affected by these limitations when interpreting study findings. Field‐based studies using portable equipment face additional constraints that should be acknowledged and, where possible, quantified through sensitivity analyses.

### 3.4 Confounding and Covariate Selection

Confounding is the central threat to causal inference in cohort studies and the fundamental reason why observational evidence, however carefully designed, cannot be treated as equivalent to evidence from an RCT. In an RCT, random assignment ensures that treatment allocation is statistically independent of all participant characteristics, both measured and unmeasured. As a result, any difference in outcomes between groups can be causally attributed to the treatment. By contrast, in a cohort study, exposure is not assigned; it arises from the complex interplay of biological, behavioural and social factors that may also determine the outcome. Individuals who develop periodontitis, for example, tend to be older, more likely to smoke, of lower socioeconomic position, and to have poorer access to preventive care than those who do not. Many of these same factors independently increase the risk of metabolic disease, cardiovascular events or adverse pregnancy outcomes. Any observed association between periodontitis and a systemic outcome is therefore a mixture of the true biological effect and the contribution of shared determinants. Even with careful measurement and sophisticated modelling, this ambiguity cannot be fully eliminated.

The selection of covariates for adjustment must be guided by explicit causal reasoning rather than statistical criteria alone [25]. Data‐driven variable selection based on statistical significance or model fit can introduce bias by adjusting for colliders, that is, variables caused by both the exposure and the outcome or their determinants, which paradoxically opens non‐causal pathways and distorts effect estimates. Directed acyclic graphs (DAGs) provide a principled framework for encoding causal assumptions, identifying the minimal sufficient adjustment set needed to block all confounding paths, and distinguishing confounders from mediators and colliders. A DAG, however, is only as credible as the causal hypotheses it encodes; a misspecified DAG will yield a misspecified adjustment set, and DAGs constructed *post hoc* to fit the available data (and results) risk rationalising, rather than guiding, the analysis [26]. Several practices help to mitigate this. The DAG should be specified before the analysis is conducted, ideally pre‐registered, and should be published alongside the results so that the underlying assumptions are open to scrutiny [27, 28]. Systematic approaches to DAG construction, such as the Evidence Synthesis for Constructing DAGs framework, draw on the existing literature rather than individual author judgement. Where reasonable disagreement remains about the causal structure, sensitivity analyses under alternative plausible DAGs allow the robustness of conclusions to be assessed [29]. Reporting results across the relevant set of specifications, rather than presenting a single preferred analysis, is increasingly recognised as a more honest representation of the evidence that observational data can support [30, 31, 32]. Even after careful DAG‐guided adjustment, residual confounding from unmeasured or imperfectly measured variables remains a persistent limitation of observational research. Sensitivity analyses such as calculation of E‐values may be reported to contextualise causal claims [33].

## 4 Limitations and Biases

As discussed, the cohort study has several important strengths relative to other observational designs. Temporal ordering of exposure before outcome is established by design, reducing the risk of reverse causation, which is a central concern in cross‐sectional studies (see Baumeister et al. this issue [34]). Multiple outcomes can be examined for a given exposure, which is particularly valuable in periodontology, given the growing evidence linking periodontal with systemic diseases [35]. Birth cohorts, in particular, enable the investigation of life course exposures and developmental programming hypotheses that are inaccessible to designs that recruit participants in adulthood.

### 4.1 Logistical Considerations

There are, however, several important limitations. The first is the logistical considerations inherent to longitudinal follow‐up. Participant data and biospecimens must be curated, maintained and remain accessible across what may be many decades, requiring dedicated physical and electronic infrastructure. Participants themselves must be retained and re‐contacted at pre‐specified assessment waves, which places sustained demands on study personnel and administrative systems. These operational requirements translate directly into cost, and deeply phenotyped cohort studies are correspondingly expensive to establish and maintain. Sample size presents a further constraint: many exposures and outcomes are uncommon, and precise estimation requires large cohorts, such that studies of rare conditions over extended follow‐up may be infeasible in practice. The logistics and financial barriers to establishing prospective cohorts are therefore substantial and, in many settings, prohibitive.

### 4.2 Right‐Censoring

The next consideration is that, in order to establish a temporal ordering, cohort studies follow a longitudinal design. That is, unlike cross‐sectional designs, there is an important time component which must be considered. The introduction of time as a key component in study design also brings to light additional limitations. These sources of bias are illustrated in Figure 4.

**FIGURE 4:**
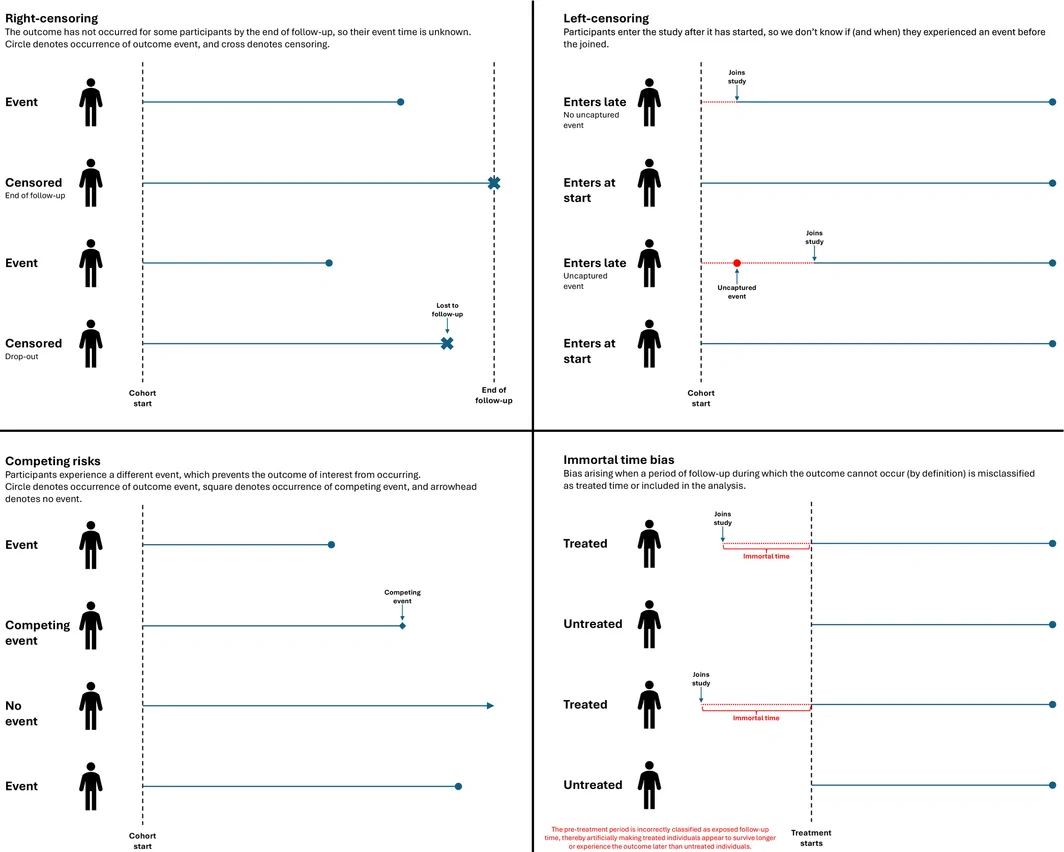
Potential sources of time‐related biases in cohort studies: Right‐censoring, left‐censoring, competing risks and immortal time biases. If censoring is unrelated to the exposure and outcome, then it is not necessarily a source of bias, but investigators will need to consider whether this is the case for their particular analysis.

The first of these time‐related sources of bias is loss to follow‐up. This can take many names, including *attrition* or *censoring*. To be precise, loss to follow‐up is a form of *right‐censoring*. This refers to the fact that, if one imagines a horizontal timeline, with enrolment into the cohort on the left and later times on the right, then loss to follow‐up represents a loss of information on the right‐hand side of this timeline. Unlike a cross‐sectional design, a cohort study depends on participants remaining observable over time, and the completeness of follow‐up is often directly related to the validity of its findings. Censoring becomes particularly problematic when the probability of dropout is related to both the exposure and outcome, which is a form of selection bias often termed *attrition bias* or *informative censoring*. In this setting, participants who remain under observation no longer represent the source population. Consequently, complete‐case analyses estimate the association among the selected subset who remain observed, which may differ from the estimand originally targeted in the full cohort. Thus, informative censoring can compromise internal validity through selection bias and may also reduce the generalisability of findings to the broader source population. This problem is compounded in long‐term periodontal cohort studies because the factors most strongly associated with dropout—including sex, socioeconomic position and health status—are also the strongest predictors of both periodontitis severity and systemic comorbidity [36]. Analytical approaches, including weighting by the inverse probability of censoring, which reweights the retained sample to recover representativeness, and tipping‐point analyses, which quantify the degree of informative censoring that would be required to explain away the observed association, should be routinely considered [37, 38].

### 4.3 Left‐Censoring

In the same way that incomplete information during follow‐up can induce bias, incomplete information before cohort entry can also threaten validity. This problem is commonly referred to as *left‐censoring*, reflecting the conventional representation of timelines in which earlier time points appear to the left. Left‐censoring arises when relevant events, exposures or disease history occurring before the start of observable data are unrecorded, leading to misclassification at baseline. For example, consider a cohort study evaluating the effect of diabetes progression on subsequent periodontal outcomes using routinely collected health records. If a participant developed diabetes several years before entering the database, but only recent records are available, that individual may be incorrectly classified as having newly diagnosed diabetes or as having a shorter disease duration than is truly the case.

The standard approach to mitigating left‐censoring is to require a minimum period of observable data before cohort entry, often termed a *look‐back period* or *wash‐in period* [3]. This pre‐entry window is intended to ensure more complete ascertainment of prior diagnoses, treatments and other clinically relevant events before follow‐up begins. Some studies require a minimum period of prior data to be available and then only use the information recorded during that fixed interval, whereas others may use all historic information once the minimum threshold is met.

### 4.4 Immortal Time Bias

The concept of *time zero*, that is, the synchronised time point in a study when eligibility criteria are met, treatment strategy is assigned, and follow‐up for the outcome begins, is central to the valid design of any cohort study [24, 39]. In an adult cohort such as SHIP, time zero corresponds not simply to the baseline examination date, but to the point at which participants meet eligibility criteria, are classified according to their baseline periodontal status, and begin follow‐up. In a birth cohort such as Pelotas, birth itself may serve as time zero for some life course exposures and outcomes; however, the relevant analytic time zero may differ depending on the specific exposure–outcome question under investigation and the point at which eligibility, exposure classification and follow‐up are aligned.

Misspecification of time zero is a common source of *immortal time bias*, where a period during which the outcome cannot occur by design is erroneously included within the follow‐up or exposure window, thereby artificially making the exposure appear protective. For example, suppose a cohort is defined as all individuals who have attended a particular healthcare facility continuously for 1 year, and subsequent outcomes are compared according to whether they received periodontal treatment during that first year. To be classified as exposed, participants must remain alive, under observation and free of the outcome long enough to receive treatment. If this initial year is then counted as exposed follow‐up time, the treated group is guaranteed a period during which the outcome could not yet have occurred by definition. Any comparison with untreated participants will therefore tend to exaggerate the apparent protective effect of treatment.

Explicit and consistent definition of time zero is, therefore, foundational to a valid cohort study design. It is particularly pertinent in pharmacoepidemiology studies when subcohorts are defined using prescriptions filled after enrollment into the study. For further information about immortal time bias, please refer to Yadav and Lewis [40].

### 4.5 Competing Risks

In periodontal cohorts with long follow‐up, competing risks, particularly tooth loss and edentulism, may preclude the occurrence of the periodontal outcome of interest. A *competing risk* is an event that either prevents the outcome from subsequently occurring or fundamentally alters the probability that it can be observed. In survival analysis, this violates the assumption that individuals who are censored remain exchangeable (i.e., comparable) with those who continue under observation. Treating competing events as ordinary non‐informative censoring can therefore lead to biassed estimates of disease incidence and exposure‐outcome associations.

This issue is especially relevant in periodontology because the structures required for outcome measurement may themselves be lost over time. Once a tooth has been extracted, subsequent probing depth progression, attachment loss or other measures of tooth‐level periodontal destruction can no longer be observed for that tooth. At the person‐level, complete edentulism removes an individual from risk of future periodontal disease altogether, while simultaneously reflecting a history of severe disease, limited access to care, smoking or other determinants that may also be related to the exposure under study. Tooth loss is therefore rarely a neutral censoring event; it is often informative and causally intertwined with the periodontal process itself. Investigators should therefore define clearly whether tooth loss and other outcomes are part of the disease process, a competing event or a composite endpoint with periodontal progression. This choice is conceptual as much as statistical, and should be driven by the question of interest. Transparent reporting of the frequency and timing of competing events, together with sensitivity analyses under alternative outcome definitions, is essential for valid inference in longitudinal periodontal cohort studies.

The combination of confounding, plus the range of aforementioned potential biases, means that even a well‐designed and carefully analysed cohort study can only approximate the counterfactual comparison that would be obtained from an RCT. Explicit acknowledgment of this limitation is essential for credible causal inference.

### 4.6 Specific Considerations in Periodontology

The aforementioned cohort designs are not equally represented in the periodontal literature. A substantial portion of the evidence base that guides contemporary practice was produced under design conventions that predate the methodological considerations discussed here, but continues to shape clinical interpretation and inform practice guidelines. This limitation is evident, for example, in the most recent S3‐level guidelines, where some of the systematic reviews used to inform practice include studies comprised almost exclusively from over 25 years ago [41]. Furthermore, in many clinical cohort studies, considerable attention is devoted to measurement reliability and clinical standardisation. While these aspects are important, methodological challenges such as confounding, selection bias and loss to follow‐up often have a greater impact on the validity of causal inference.

One particular type of study arises frequently in the periodontal literature, namely when a single group of patients receiving a defined intervention is followed over time with outcomes such as tooth survival, pocket depth reduction or implant survival reported. The studies are often labelled prospective cohort studies. In this setting, the distinction between what constitutes a case series versus a cohort study is subtle, but important [42, 43]. A cohort study is defined with respect to an exposure of interest; that is, exposed individuals are included on the basis of a shared intervention, disease, characteristic or behaviour [42, 43]. Most cohort studies include a comparator (i.e., unexposed) group, whereas case series do not. However, the inclusion of a comparison group is not a defining feature of a cohort study [42, 43]. Therefore, it is possible for a study to include a group of individuals who all receive the same periodontal therapy and still be appropriately considered a cohort study.

What differentiates a cohort study from a case series is the *sampling frame* and the underlying *purpose* of participant selection. In a cohort study, participants are defined at a clear starting point (time zero) on the basis of exposure status, and are followed longitudinally to observe the occurrence or timing of outcomes in relation to that exposure. Even when only a single exposed group is present, the study remains a cohort if it represents a defined population at risk that is followed forward in time. In contrast, a case series is primarily descriptive. Participants are typically assembled because they received a particular treatment or share a clinical condition, without a clearly defined at‐risk population or an explicit framework for comparing outcome occurrence across exposure groups.

Consider a study in which all patients receiving non‐surgical periodontal therapy in a specialist clinic over a 1‐year period are enrolled and followed to assess tooth loss and disease recurrence. Because participants are defined at a clear time zero (treatment initiation) and followed longitudinally as a defined group at risk of subsequent outcomes, this design is consistent with a cohort study, even in the absence of an untreated comparator group. The population to which the inferences apply is well‐defined; it is the population of all individuals attending the given specialist clinic.

In contrast, consider a clinical report describing 50 patients who underwent the same regenerative periodontal procedure, with outcomes such as pocket depth reduction and radiographic bone fill reported. Because there is no defined sampling frame, and the study represents a convenience sample of all individuals receiving the treatment of interest, it is not clear whether this reflects a defined source population. These 50 patients may or may not be representative of all patients undergoing the same procedure in that clinic, let alone in other settings. In this setting, the design is more appropriately characterised as a case series rather than a cohort study.

Consider a further borderline example involving an implant maintenance programme in which all patients with dental implants placed within a national registry are followed annually for implant survival and peri‐implant disease. Although no untreated comparator group is present, this design may still be considered a cohort study if the registry defines a clear population at risk (all implant recipients), establishes a time zero (implant placement or prosthetic loading) and systematically follows individuals over time for outcome occurrence. However, if the registry data are incomplete, opportunistic or primarily compiled from follow‐up visits without a clearly defined denominator of all eligible implant recipients, the study may drift toward a descriptive case series, despite being labelled a cohort study. It is no longer clear who the source population is.

Whilst the difference may initially appear to be minor, the distinction between a case series and a cohort study should not be treated as purely semantic. A well‐defined cohort allows inference to a clearly specified source population, whereas a case series generally does not support such inference beyond the described patients. Also, whilst some single‐arm periodontal studies may meet the formal criteria for a cohort, they may remain close to a case series in the type of inference they support. This may occur when the study population is defined only after substantial clinical selection has taken place, or when inclusion is restricted to patients treated by a single clinician, retained in long‐term follow‐up and documented with complete records. In such circumstances, the study may describe outcomes within a highly selected clinical experience rather than estimate prognosis for a broader population of patients undergoing the treatment. Therefore, an important step for investigators designing and reporting single‐arm periodontal treatment studies is to be explicit about the sampling frame, the definition of time zero, the process by which participants entered the study, and the target population to which the findings are intended to generalise.

### 4.7 Clinical Implications

Although the present article focuses on methodological issues in cohort study design, these considerations have direct implications for clinical practice in periodontology. Observational cohort studies are frequently used to inform treatment recommendations, particularly where randomised controlled trials are unavailable or impractical. However, as illustrated in the preceding sections, investigators must ensure that several sources of bias are correctly handled.

For clinicians, the key implications centre around the fact that observational evidence should not be discounted, but that its interpretation requires careful attention to study design, especially how the cohort was assembled, how time zero was defined, and whether the estimated effect corresponds to a clearly defined target population (Box 2). Detailed, transparent reporting of these elements is crucial for the translation of epidemiologic evidence into more reliable and clinically meaningful treatment recommendations (Figure 5).

BOX 2 **Summary of key sources of bias in cohort studies.**

**Table:** 

**Population** If the study population is not clearly defined or is not representative of the target clinical population, results may not be generalisable to routine periodontal practice
**Sampling** Non‐random or convenience sampling can lead to systematic differences between study participants and the broader patient population
**Measurement** Imprecise or inconsistent measurement of exposures or outcomes can lead to misclassification, which may dilute true associations or create spurious ones
**Confounding** If important risk factors are not adequately accounted for, observed treatment effects may reflect differences in patient characteristics rather than true causal effects
**Censoring** Loss to follow‐up can distort results if dropout is related to both exposure and outcome, leading to misleading estimates of treatment effectiveness
**Time zero** If time zero is not correctly aligned this can result in misleading conclusions, such as the exposure appearing to be protective, when it is not
**Competing risks** Failure to account for events such that preclude the outcome of interest, for competing risks may overestimate or underestimate true incidence
**Logistics** Cohort studies are expensive and practical constrains may limit the quantity and quality of data available

**FIGURE 5:**
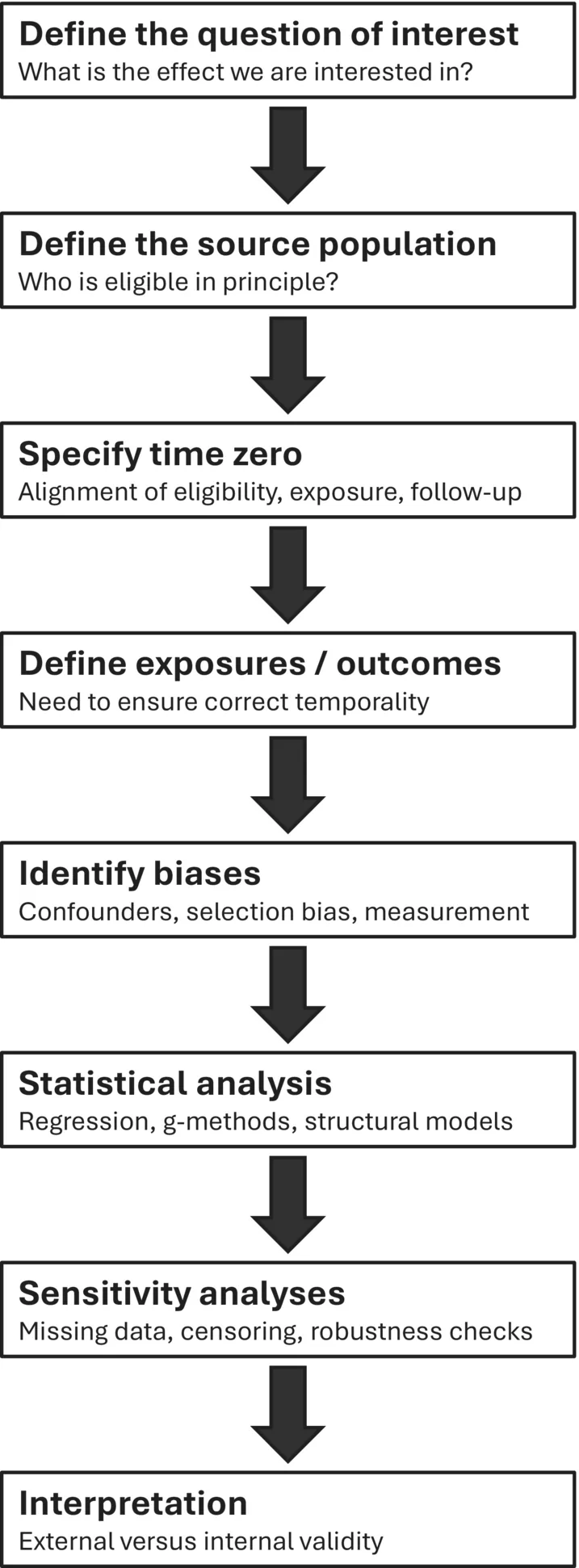
Illustration of the key steps in carrying out a cohort study.

In addition, the increasing use of large‐scale routinely collected datasets and artificial intelligence methods in periodontology warrants brief consideration. Machine learning approaches and automated data extraction techniques can facilitate the analysis of very large cohorts derived from electronic health records, administrative databases and imaging or radiographic repositories. These developments offer important opportunities for identifying patterns of disease, predicting outcomes and scaling observational research to populations that would be infeasible to study using traditional designs. However, the fundamental principles outlined in this article remain unchanged. Regardless of whether exposure and outcome variables are derived through manual abstraction or artificial intelligence algorithms, valid inference still depends on a clearly defined target population, appropriate specification of time zero, adequate handling of confounding and careful consideration of aforementioned biases. Artificial intelligence, therefore, does not alter the underlying requirements of cohort design but rather changes the scale and complexity at which these design principles must be applied.

## 5 Addressing Biases by Design

### 5.1 Collider Bias and Index Event Bias

Consider a study exploring the determinants of long‐term tooth loss, in which the investigator restricts the analysis to individuals with complete dentition (i.e., no prior tooth loss) at baseline. Both periodontitis and dental caries are established causes of tooth loss. Conditioning on the absence of prior tooth loss, therefore, amounts to conditioning on a common outcome (i.e., a collider) of these two conditions. In other words, among individuals with complete dentition at baseline, the presence of one condition (e.g., periodontitis) implies a lower likelihood of the other (e.g., dental caries), even if they are independent in the source population. This could create the misleading impression that the two conditions are ‘protective’ against one another. As a result, this induced association between periodontitis and dental caries may bias estimates in any downstream analyses. This is illustrated in Figure 6.

**FIGURE 6:**
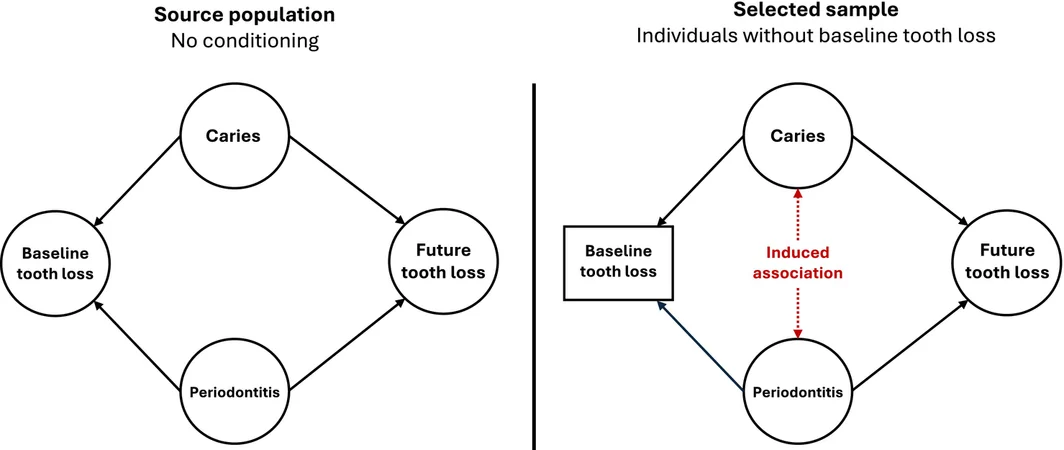
Directed acyclic graph illustrating the structure of index event bias in a hypothetical study investigating the effect of periodontitis and dental caries on tooth loss. Arrows indicate causal relationships, with arrow direction representing the direction of effect. Circular nodes represent variables of interest. Square nodes represent variables that have been conditioned on (e.g., by restricting the analysis to a specific level or by statistical adjustment). Red dashed lines represent correlations between variables without necessarily implying causation. Left: In the source population, periodontitis and dental caries may be independent causes of tooth loss. Right: After restricting the analysis to individuals with complete dentition (i.e., no tooth loss at baseline), a correlation is induced between periodontitis and dental caries, even if they share no causal relationship. This induced correlation may bias downstream analyses of disease progression.

More generally, restricting analyses according to a prior outcome or intermediate event can distort exposure–outcome relationships by inducing non‐causal associations. When this occurs because inclusion into the study sample is determined by some prior event related to both the exposure and outcome, this prior event is referred to as an *index event*, and the resulting selection bias is often termed *index event bias*. Index event bias may therefore be understood as a specific manifestation of collider bias that may arise in epidemiological studies.

### 5.2 The New‐User Design

In effect, cohort studies evaluating the effects of a treatment or exposure on health outcomes face a very similar challenge when the analytical sample includes individuals already using the treatment of interest at the time of study entry. These prevalent users represent a biologically selected group that differs systematically from treatment‐naive individuals, a form of selection bias termed *prevalent user bias*, that distorts comparative estimates because long‐term survivors under treatment are over‐represented relative to those who initiated treatment more recently. The new‐user design addresses this by restricting the analytical cohort to individuals initiating treatment for the first time at a well‐defined time zero [44]. A dental application by Leiva‐Escobar and colleagues demonstrated the consequences of neglecting this principle: in a study of thiazide monotherapy and dental caries risk using electronic health records, a properly specified new‐user emulation showed no significant difference between treatment groups, while a naive cohort including prevalent users suggested a harmful effect of thiazide [45]. The difference in findings arose entirely from the biologically selected survivor population retained among prevalent users, not from any underlying pharmacological effect.

### 5.3 Target Trial Emulation

The target trial emulation framework builds on some of the principles seen in the new‐user design by providing a structured protocol for designing observational cohort studies as explicit emulations of the hypothetical RCT that would most directly answer the causal question of interest [46]. Investigators prespecify seven core components: eligibility criteria, treatment strategies, treatment assignment, time zero, outcomes, causal contrast and statistical analysis plan, then map each onto available observational data. This structured approach compels explicit articulation of the research question and prevents the design errors that generate bias in conventional observational analyses.

The Leiva‐Escobar et al. study [45] provides a concrete dental illustration. Beyond the new‐user and prevalent user cohorts, the authors constructed a nonaligned cohort in which follow‐up began at the date of hypertension diagnosis rather than at treatment initiation, erroneously counting the treatment‐free interval as exposed time. This induced immortal time bias and produced an effect estimate directionally opposite to the prevalent user cohort, despite using essentially the same data. The reversal of effect direction across the two naive cohorts illustrates how design‐related biases can yield results that are not only quantitatively wrong but qualitatively misleading, and underscores why the explicit alignment of eligibility assessment, treatment assignment and follow‐up start at a common time zero is foundational to valid causal inference from observational data. A dedicated article in this series addresses target trial emulation in depth [24].

## 6 Addressing Biases by Analysis

Once a cohort has been defined and follow‐up established, the next step is translating the observed data into an estimate of effect that answers a clinically meaningful question. Different statistical approaches answer slightly different questions about patient outcomes. A useful distinction is between *how often* something happens over time and *how quickly* it happens at any given moment. The first is closer to a *risk over a period of follow‐up* (e.g., ‘what proportion of patients lose a tooth within 10 years?’), while the second is closer to a *rate at a point in time* (e.g., ‘how quickly is tooth loss occurring among those still at risk?’).

Cox proportional hazards models (Box 3) are commonly used by cohort studies, which produce *hazard ratios*. Although widely reported, this measure does not directly tell us how much more likely tooth loss is over the entire study period. Instead, it compares the instantaneous rate at which events occur between groups. This can be misleading in periodontal settings where events are relatively common and occur over long follow‐up periods, because a faster early rate does not necessarily translate into a proportionally higher long‐term probability of tooth loss. For clinicians, a more intuitive question is often, ‘What is the difference in the chance of tooth loss over time between groups?’ This type of question is better aligned with risk‐based summaries, which describe how many patients experience the outcome during follow‐up. In many datasets, hazard ratios and risk differences will not tell exactly the same story, especially when disease progression accumulates over many years, or when follow‐up is long. A related issue concerns the widespread use of logistic regression and the reporting of odds ratios in dental research. Odds ratios are appropriate effect measures in case–control designs, or when the outcome is rare. However, many outcomes of interest in dentistry are common, whether expressed as disease states (such as periodontitis) or as treatment outcomes (such as resolution of peri‐implantitis), and the odds ratio may systematically overstate the risk. For cohort studies with time‐to‐event outcomes, models that explicitly account for follow‐up time are generally preferable to logistic regression, and effect measures should be chosen and interpreted accordingly.

BOX 3 **Summary of the principles of survival analysis.**

**Table:** 

**What is survival analysis?** A set of methods used to analyse the time until an event occurs, such as tooth loss, implant failure or mortality It incorporates when events happen, not just if they happen **What is a time‐to‐event outcome?** Each participant contributes a time from a defined starting point to either an event (e.g., tooth loss) or censoring (e.g., end of follow‐up without event) **What is censoring?** Left: Occurs when the event has already happened before observation begins, so the exact event time is unknown e.g., tooth loss occurred before baseline assessment Right: A participant does not experience the event during the observed follow‐up period, so the exact event time is unknown beyond the last time they were observed Interval: Occurs when the event is known to have happened between two observation time points, but the exact timing is unknown **What is a survival curve (Kaplan–Meier curve)?** A Kaplan–Meier curve shows the probability of remaining event‐free over time in a defined cohort The curve starts at 100% at time zero and steps downwards each time an event occurs **How are survival curves constructed?** At each event time, the probability of surviving beyond that time is updated based on the number of individuals still at risk Censored individuals are removed from the risk set but do not cause a drop in the curve **How should survival curves be interpreted?** Survival curves describe the observed time‐to‐event experience of a cohort They show when events occur, but do not by themselves imply causality between exposure and outcome **What does survival analysis allow us to do?** Allows comparison of event timing between groups Provides estimates such as survival probabilities and hazard ratios.

There are two main approaches to handle competing events: *cause‐specific* hazard models and *subdistribution* hazard models [47, 48, 49]. Cause‐specific hazard models ask, ‘Among patients who are still event‐free at a given time, how does the instantaneous rate of the event of interest differ between groups?’ In periodontal terms, this is closest to focusing on the biological process leading specifically to tooth loss, while treating other events (such as death or extraction for non‐periodontal reasons) as ending follow‐up. This approach is particularly useful for understanding aetiological relationships because it isolates the process that directly generates the outcome of interest. However, it does not directly translate into the overall probability that a patient will experience tooth loss over time. By contrast, subdistribution hazard models focus on the question, ‘What is the probability that a patient will experience tooth loss over time in the observed setting where other events can occur?’ Rather than removing individuals who experience competing events, this approach retains them in a modified way so that the estimated effect reflects how outcomes accumulate in a population. As a result, subdistribution hazard models are often more aligned with prognosis and patient counselling, where the primary interest is the overall chance of tooth loss over a given follow‐up period. In periodontal research, these two approaches can lead to different interpretations. A risk factor may strongly influence the biological process of periodontal breakdown (cause‐specific effect) but have a more modest impact on the overall probability of tooth loss once competing risks are considered, or vice versa. The choice between them should therefore be guided by whether the aim is to understand disease mechanisms or to estimate risk.

Confounding is addressed through methods designed to make exposure groups comparable with respect to measured baseline characteristics. The most common approach remains multivariable regression adjustment, where variables such as age, smoking, diabetes and baseline periodontal severity are included in the model. Propensity score methods take a different approach by explicitly constructing comparable groups based on the likelihood of receiving a particular exposure or treatment [50, 51]. This can be achieved through matching or weighting and is particularly useful when treatment allocation is strongly influenced by clinical characteristics. Standardisation‐based methods, such as g‐computation, go further by directly estimating what would happen if everyone in the cohort were exposed versus if no one were exposed. This produces population‐level comparisons that are often easier to interpret clinically than model‐dependent contrasts [52]. Such approaches can also be adapted to allow for time‐varying exposures, outcomes and covariates which are time‐varying [53, 54]. A further class of methods, causal mediation analysis, is particularly well suited to the life course questions that birth cohort designs make tractable. When the goal is to decompose effects into components operating through specific pathways, mediation methods explicitly separate the *direct effect* of an exposure from the portion transmitted through intermediate variables [55, 56]. This is especially relevant in periodontal research, where many exposures of interest (such as socioeconomic status or early‐life conditions) operate through multiple downstream behavioural and biological pathways.

When censoring, whether left‐ or right‐censoring, is thought to be related to the underlying risk of the outcome of interest, then specific methods may be required. This is plausibly the case in periodontal cohorts where dropout may be related to disease severity, dissatisfaction with care, socioeconomic constraints or worsening oral health. In the case of right‐censoring, one common approach is inverse probability of censoring weighting, which re‐weights individuals who remain under observation so that they also represent those who were lost [57]. This helps reduce bias when dropout is more common in higher‐ or lower‐risk patients. Analytical approaches for addressing informative tooth loss also remain underused in periodontal research, and inverse probability of censoring weighting may be extended to the tooth level, where patient‐level weights are derived from tooth‐level models to account for the fact that teeth at higher risk of loss are also those most informative about disease progression [58]. For left‐censoring, the key requirement is to correctly align follow‐up time so that individuals only contribute person‐time from when they become observable, conditional on having remained event‐free up to that point. This avoids preferentially excluding early failures and is typically handled using risk‐set formulations of survival models. Together, these approaches ensure that time at risk is correctly defined and that selective loss of participants does not distort estimates of periodontal progression or tooth loss.

There are also a number of other considerations, including accounting for the correlation between repeated measures within individuals, issues due to measurement error in clinical assessments, heterogeneity in follow‐up intervals and clustering of patients within clinicians or treatment centres. Each of these features can influence the bias, precision and interpretation of effect estimates. Taken together, these points highlight that investigators can deploy a wide range of analytical approaches to the same periodontal cohort, each corresponding to a different way of representing time, disease processes and patient experience. The choice of method, therefore, determines what the resulting estimate is actually describing.

## 7 Comparison With Case–Control Designs

The *case–control* design is another widely used type of study design in clinical research. In a case–control study, participants are sampled according to outcome status rather than exposure status. Individuals with the disease or outcome of interest (cases) are identified from a defined source population, and a sample of individuals without the outcome (controls) is selected from the same underlying population [3]. The fundamental principle is that controls should represent the exposure distribution in the population that gave rise to the cases and should be selected independently of the exposure under study. Unlike cohort studies, which begin with a population at risk and follow individuals forward to observe outcome occurrence, case–control studies sample on the outcome and then compare prior exposures between cases and controls. Consequently, the validity of a case–control study depends critically on the clear definition of the source population and the unbiased selection of both cases and controls from that population.

Several variants of the case–control design exist, differing mainly in the way controls are selected from the source population. Controls may be sampled from individuals at risk when each case occurs (density or risk‐set sampling), from the cohort at baseline (case‐cohort sampling), or from individuals who remain disease‐free at the end of follow‐up (cumulative sampling) [3]. Although these approaches target different epidemiologic measures, they share the same underlying logic: a sample of controls is used to represent the exposure experience of the population that gave rise to the cases.

Many of the principles governing participant selection in cohort studies apply equally to case–control studies [3]. In both designs, valid inference depends on a clearly defined source population and transparent eligibility criteria. Case–control studies ideally include incident rather than prevalent cases, as prevalent cases may reflect not only disease occurrence but also factors influencing disease duration or survival. Likewise, the objective is not necessarily to obtain a sample that is representative of the general population, but rather to ensure that cases and controls arise from the same underlying source population. In this respect, concerns regarding selection, eligibility and the target population of inference are largely shared between cohort and case–control designs.

A common misconception is that case–control studies are inherently more susceptible to bias or provide weaker evidence than cohort studies. In reality, a properly designed case–control study can be viewed as an efficient sample from an underlying cohort and, when cases and controls are selected appropriately from the same source population, can provide valid estimates of the same underlying associations. Many concerns commonly attributed to case–control studies, such as recall bias, selection bias and misclassification of exposure, are not unique to the design itself but arise from how data are collected and participants are identified. Similarly, assumptions that controls should be representative of the general population, restricted to healthy individuals or limited to those with an opportunity for exposure reflect misunderstandings of the design. The central requirement is that controls represent the population that gave rise to the cases. Consequently, many of the methodological challenges encountered in case–control studies are shared with cohort studies, including accurate exposure measurement, appropriate timing of classification, control of confounding and avoidance of selection bias [3]. A particular misapplication of the case–control design that deserves attention in periodontology is the comparison of individuals with periodontitis against those without the condition, labelled as ‘cases’ and ‘controls’ respectively. This framing conflates a case–control study with a simple case versus non‐case comparison and does not satisfy the fundamental requirements of the design: a defined source population, a valid sampling frame for controls, and an exposure of interest whose distribution is being compared.

A closely related misapplication, particularly common in the implant literature, mistakes the sampling axis altogether: groups are defined by an exposure rather than by the outcome—for example, comparing marginal bone loss or implant survival between patients with and without diabetes, or between smokers and non‐smokers, with the exposed group designated as ‘cases’ and the unexposed as ‘controls’. Whereas the periodontitis comparison at least samples on disease status, here the investigator samples on the exposure and follows the groups forward to the outcome [59]. In its commonest form, the two exposure groups are matched and then followed over several years for implant survival and marginal bone loss, with the study nonetheless described as a ‘case–control study’—a retrospective cohort in all but name. This distinction is not merely semantic: a methodological review of the implant literature found that among 104 full‐text studies on smoking, diabetes or periodontitis and implant loss, only three qualified as cohort studies and none as case–control studies; most were case series or cross‐sectional studies [60]. The dual meaning of the term ‘control’—an unexposed comparison arm in a cohort or trial, but a non‐diseased subject sampled from the source population in a case–control study—may be the cause of this conflation. Furthermore, given that periodontitis is a common condition worldwide, the use of the case–control design is rarely justified on efficiency grounds, since the design is intended for rare outcomes. Researchers should therefore exercise caution when applying this label and consider whether a cross‐sectional or cohort approach would be more appropriate for the research question at hand.

Despite their similarities, cohort and case–control studies differ in important practical respects (Table 3). Cohort studies begin with a population at risk and follow individuals over time to observe outcome occurrence, allowing direct estimation of incidence, risks, rates and absolute measures of effect. They are particularly well‐suited for studying multiple outcomes associated with a single exposure and for establishing the temporal sequence between exposure and outcome. However, cohort studies may require large sample sizes, prolonged follow‐up and substantial resources, particularly when outcomes are rare or have long latency periods. In contrast, case–control studies begin with outcome occurrence and are generally more efficient for investigating rare diseases or outcomes that take many years to develop. Because only a sample of the source population is studied, they often require fewer resources and can be conducted more rapidly. Their principal challenge lies in the identification of an appropriate source population and the unbiased selection of controls. Thus, neither design is inherently superior; rather, the choice between a cohort and case–control design should be guided by the research question, the frequency of the outcome, the availability of data and practical considerations of feasibility and cost.

**TABLE 3:** Comparison of cohort and case–control study designs.

Consideration	Cohort study	Case–control study
Sampling basis	Exposure or population at risk	Outcome or disease status
Temporal direction	Forward in time	Retrospective (from outcome)
Measures	Incidence, risk, rate	Odds ratio (as an estimator of the RR or IRR, depending on the design)
Efficiency	Less efficient for rare outcomes	More efficient for rare outcomes
Follow‐up	Required	Not required
Key strengths	Addresses multiple outcomes and temporality	Addresses rare outcomes and efficiency (i.e., time and cost)
Key limitations	Cost, time, attrition	Source population and selection issues
Main bias concerns	Loss to follow‐up and time‐varying confounding	Selection bias and recall bias

Abbreviations: IRR, incidence risk ratio; RR, risk ratio.

## 8 Conclusions

Cohort studies are indispensable tools in modern health research, and periodontology is no exception. Because they can guarantee temporal ordering, they allow for lines of inquiry pertaining to the determinants and distribution of disease incidence, prognosis, and its longitudinal effects. The designs discussed in this article, from prospective birth cohorts with specifically designed sampling and phenotyping protocols, through to retrospective cohorts which arise from convenience samples, have the potential to address a unique set of scientific questions. The choice of study design is not merely a logistical matter but a fundamental methodological decision that shapes which biases can be avoided, which estimand can be targeted, and which analytical tools can be deployed. No single design is universally superior, as the appropriate choice depends on the research question, the nature of the problem and the resources available to the investigator.

Methodological advances, both in terms of study design and statistical methods, have raised the bar for what constitutes a credible cohort study. The design and analytical considerations reviewed here reflect the evolution of contemporary observational research, where increasingly sophisticated tools are available to address potential sources of bias. Their application should not be viewed as a methodological luxury, but as an essential requirement if the evidence generated from cohort studies is to justify the influence it holds over clinical practice, guideline development and policy.

## Funding

N.Z.B. is supported by the Wellcome Trust (322777/Z/24/Z). N.Z.B. is a member of the Medical Research Council Biostatistics Unit (MC_UU_00040/01). J.R.H.T. is supported by the Singapore National Medical Research Council (NMRC) Research Training Fellowship (RTF24jan‐0007). J.B. is supported by a national fund of the Fundação para a Ciência e Tecnologia (https://doi.org/10.54499/UIDB/04585/2025).

## Conflicts of Interest

The authors declare no conflicts of interest.

## Data Availability

Data sharing is not applicable to this article as no datasets were generated or analysed during the current study.
